# Timely linkage of individuals to substance use disorder treatment: development, implementation, and evaluation of FindHelpNowKY.org

**DOI:** 10.1186/s12889-019-6499-5

**Published:** 2019-02-11

**Authors:** Terry Lee Bunn, Dana Quesinberry, Tyler Jennings, Amber Kizewski, Heather Jackson, Sarah McKee, Sarah Eustice

**Affiliations:** 1 Kentucky Injury Prevention and Research Center, University of Kentucky, 333 Waller Ave., Suite 242, Lexington, KY 40504 USA; 2 Department of Preventive Medicine and Environmental Health, University of Kentucky, College of Public Health, 111 Washington Ave, Lexington, KY 40536 USA

**Keywords:** Substance use disorder, Treatment, Access, Partnerships, Development, Implementation, Evaluation, Website, Addiction

## Abstract

**Background:**

Substance use disorders (SUD) have steadily increased over the last two decades. Seeking SUD treatment involves searching SUD treatment facility types (inpatient, outpatient and intensive outpatient, residential and family residential, and detoxification facilities) that offer specialized SUD treatment depending on individual needs and preferences. Referrals to SUD treatment require innovative strategies that rapidly link individuals to SUD treatment when they are at the critical stage of readiness. The aim of this study was to develop, implement, and evaluate a user-friendly SUD treatment facility opening availability website called FindHelpNowKY.org. The objectives of the study were to 1) recruit SUD treatment facility and partner participation; 2) develop platform, content, and analytics for the FindHelpNowKY.org website intervention with an information repository; 3) assess barriers and facilitators to implementation; and 4) evaluate the development and implementation of FindHelpNowKY.org.

**Methods:**

Website development stakeholders were identified and the website concept was developed. The logic model for FindHelpNowKY.org outlined resources, activities, and outputs as well as the associated short-term, medium-term, and long-term objectives, along with a website evaluation plan. Website usability and focus group testing was conducted. Information repository resource documents were compiled and categorized. An inventory of Kentucky-based SUD treatment facilities was compiled using various state and federal resources.

**Results:**

Development/implementation barriers were addressed, facilitators were identified, and the website was implemented; 83% of SUD treatment facilities were indexed on the website, and average website user time was 7 min. From February to October 2018, there were 29,000 visitors, and 30,000 unique searches. The most common website query was a friend or family member seeking long-term residential or outpatient treatment facilities accepting Medicaid or Medicare.

**Conclusions:**

FindHelpNowKY.org has the potential to fill a critical need for timely access to available SUD treatment in the state. The website may be a valuable resource for health professionals that can enhance clinical workflow and reduce staff time conducting phone and website searches for available SUD treatment. The website is a promising tool for assessing current SUD treatment capacity vs. SUD treatment need. The FindHelpNow model can be used by other states to increase timely access to SUD treatment.

## Background

Globally, the number of individuals with substance use disorders (SUD) has steadily increased over the last two decades. The *World Drug Report 2017* estimates that in year 2015, 29.5 million people had an SUD, comprising 0.6% of the adult population worldwide [1]. In the United States, it was estimated that in 2016, over 20 million people had a SUD, and 7.4 of the 20 million had an illicit drug use disorder [2]. Only about 10% of individuals with SUDs receive any type of specialty treatment [3]. Kentucky had the 6th highest drug overdose fatality rate in the nation in 2016 according to National Center for Health Statistics website statistics, and had the 3rd highest rate of opioid use disorder among females who gave birth at hospital [4]. These staggering international, national, and state morbidity and mortality numbers and rates of individuals with substance use disorders require available substance use disorder treatment.

Seeking SUD treatment involves searching SUD treatment facility types for specialized treatment depending on individual needs and preferences; SUD treatment facility types include inpatient, outpatient and intensive outpatient, residential and family residential, and detoxification facilities [5]. Inpatient and residential treatment facilities may treat individuals either on a long-term basis or on a short-term basis, and typically involve detoxification first then intensive treatment. Intensive outpatient treatment allows the individual to live at home with concentrated treatment sessions then after a time, the individual can move to outpatient treatment.

There may be multiple factors that influence accessibility to SUD treatment. Barriers to SUD treatment include financial barriers such as insurance coverage status, and transportation to and from a SUD treatment facility [6, 7]. Service barriers to SUD treatment also exist, such as the need for co-treatment for other medical disorders and conditions (mental health, pregnancy, etc.) [8–10]. In addition, the availability of medication- assisted treatment (MAT) provided by MAT data waivered physicians may be a barrier for receiving evidence-based SUD treatment due to limited numbers of MAT DATA waivered physicians, limited acceptance of patients with Medicaid, and limited states with expanded comprehensive MAT coverage [11, 12]. There is also stigma and a lack of knowledge on where to go for SUD treatment [6]. The Affordable Care Act enacted in 2010 has helped to ease some of the barriers (especially financial barriers), and to increase access to SUD treatment but barriers still exist [13–15].

SUD treatment referral programs and practices require innovative strategies to rapidly link individuals to SUD treatment when they are at the critical stage of readiness. The Angel program launched by the Gloucester MA Police Department uses a voluntary screening and phone referral service within the police department without fear of arrest, although individuals that enter the police department Angel program cannot have active arrest warrants or medical or safety issues [16, 17]. In the evaluation of the Angel program, one-third of Angel program participants were placed in inpatient/residential SUD treatment and one-quarter received outpatient counseling; approximately 37% were abstinent at 6 months post SUD treatment referral. The intervention was very successful in linking individuals to SUD treatment facilities and detoxification but was limited due to the overall SUD treatment system that focuses on acute treatment, not long-term recovery. Other innovative SUD treatment referral programs have been implemented based on syringe exchange and harm reduction programs [18]. Although the harm reduction referrals to buprenorphine doctor programs were low, the authors suggested monetary and treatment cost incentives may increase SUD treatment initiation.

Web-based technology has been used to support SUD treatment and recovery interventions and practices [19, 20]. In a criminal justice-involved population, a motivational computer intervention was reported to increase SUD treatment initiation compared to standard probation intake at 2 months follow-up and approached significance at 6-month follow-up [21]. A secondary data analysis of SAMHSA Treatment Episode Datasets-Discharge (TEDS-D) data showed that SUD treatment referral was primarily through self-referral of the individual with the SUD, and through criminal justice; health care professionals and alcohol and other community referrals accounted for few referral sources [22]. While the method of SUD treatment referral was not mentioned (phone, personal, or web-based) in the study, the referral populations may benefit from an easily accessible SUD treatment availability website.

A user-friendly straightforward website referral intervention that visually displays SUD treatment facilities with available openings can improve clinical workflow and increase timely access to SUD treatment. The aim of this study was to develop, implement, and evaluate a user-friendly SUD treatment facility opening availability website called FindHelpNowKY.org. The objectives of the study were to 1) recruit SUD treatment facility and partner participation in the development of the FindHelpNowKY.org website; 2) develop platform, content, and analytics for the FindHelpNowKY.org website intervention with an information repository; 3) assess barriers and facilitators for FindHelpNowKY.org implementation; and 4) evaluate the development and implementation of FindHelpNowKY.org.

## Methods

### Website development stakeholders and website development concept

Operation Unlawful Narcotics Investigations, Treatment and Education (Operation UNITE), created by Congressman Harold Rogers in Kentucky’s Fifth Congressional District in 2003, combines law enforcement, SUD treatment, and education in Kentucky’s Appalachian counties. Operation UNITE operates a confidential toll-free Treatment Referral Line for anyone seeking assistance for a SUD including available treatment programs in the region, and programs to meet individual needs. Operation UNITE treatment program referrals include short- and long-term treatment, and recovery programs. Low-income individuals living in Kentucky’s Fifth Congressional District may also qualify for a voucher program to help cover costs of residential SUD treatment.

The Kentucky Injury Prevention and Research Center (KIPRC) held Informal planning meetings with Operation UNITE and other partners including the Kentucky Office of Drug Control Policy (ODCP), primary care providers, and other drug overdose prevention partners to identify gaps between SUD treatment capacity and SUD treatment need. The major gap that was identified was quick access to SUD treatment. To fill the gap, the concept of a SUD treatment locator, FindHelpNowKY.org, containing near real-time treatment opening availability was developed. Stakeholders also suggested available search options within the website including individual insurance coverage, SUD facility type, and the geographical distance of the nearest and furthest SUD treatment facilities (toggled by individual preference). In addition, the inclusion of a statewide help line was suggested by the planning group for inclusion in FindHelpNowKY.org development and implementation; the inclusion of a statewide help line was supported by additional funding that Operation UNITE received in 2017 to expand their regional SUD treatment referral service into a statewide help call center called KY HELP.

The logic model for the development and implementation of FindHelpNowKY.org is shown in Table 1. The long-term goal of FindHelpNowKY.org is to increase referrals to SUD treatment facilities throughout the state by health professionals and the general public. Short-term, medium-term, and long-term objectives were developed on multiple inputs: need, planned resources, activities, and outputs. Four full-time staff members were hired for FindHelpNowKY.org: three staff members were hired to identify and personally contact the SUD treatment facilities to gain agreement for participation in FindHelpNowKY.org, and one staff member was hired to work with a contractor on the physical development of the website. A fifth staff member’s effort (approximately 50%) was dedicated to website focus group testing and evaluation of the website including the development of performance indicators linked to the short-term, medium-term, and long-term objectives.

**Table 1:** Logic Model for FindHelpNowKY.org

				Outcomes		
Need	Resources/input	Activities	Outputs	Short-term (1–2 yrs)	Medium-term (3–5 years)	Long-term (< 5 years)
SUD treatment waiting time Primary care provider SUD treatment opening search time Primary care physician-SUD patient clinical workflow Lack of knowledge on SUD treatment and availability	Funding - Centers for Disease Control and Prevention (CDC) National Center for Injury Prevention and Control (NCIPC) Hired Staff Identify and contact - SUD treatment facilities - Web developer - Single state agency authority on SUD treatment facilities - Information repository collaborator Website Developer - User-friendly website - Section 508 accessibility compliant - Website access on any device Partners - KIPRC - DPH - ODCP - Operation UNITE - SUD treatment facilities - CHFS	- Collaborate with stakeholders and partners - Contact SUD treatment facilities - Develop FindHelpNowKY.org SUD treatment facility application form - Develop FindHelpNowKY.org information repository content - Design and develop FindHelpNowKY.org prototype - Focus group testing of FindHelpNowKY.org - Establish website Search Engine Optimization (SEO) capabilities - Develop website analytics modules - Implement FindHelpNowKY.org intervention statewide - Collect website evaluation data, analyze, and report results	- Availability of FindHelpNowKY.org online - Promote FindHelpNowKY.org to health professionals, and general public - FindHelpNowKY.org focus group and evaluation results - Development of 30 one-pagers on information related to SUDs	- Increased SUD help line coverage area from regional to statewide - Increased healthcare professional use of FindHelpNowKY.org - Increased collaboration among partners - Increased clicks/visitors to FindHelpNowKY.org website website after launch • Guided searches completed • Visitors to resource library - Increase comprehensive strategic alignment among statewide stakeholders to reduce SUD	- Increased treatment referrals for substance use disorders by primary care, EDs, community mental health centers, and other health professionals - Increased self-, and family- referrals to SUD treatment - Increased understanding SUD treatment options available in Commonwealth by treatment type and region Decreased time spent by healthcare professionals searching for available treatment options for patients/clients	- Increased referrals to SUD treatment facilities throughout state by healthprofessionals and the general public

### SUD treatment facility stakeholders

SUD treatment facility contacts were identified through multiple sources. The SAMHSA Behavioral Health Treatment Services Locator provided an initial list of SUD treatment facilities; the SAMHSA locator does not contain information on treatment opening availability. Other sources for SUD treatment facility contacts included the Kentucky’s Department of Behavioral Health, Developmental and Intellectual Disabilities (DBHDID) Alcohol and Other Drug Entity and Behavioral Health Services Organization (AODE/BHSO) licensure list. Staff members also attended regional community meetings attended by SUD facility representatives. Actively prescribing MAT DATA-waivered providers were identified through review of prescription drug monitoring dispensing data and linkage of active prescribers to their official address through their licensure with Drug Enforcement Agency by program staff at Kentucky’s prescription drug monitoring program.

### Website development

The FindHelpNowKY.org development contract was awarded to an independent website developer to create a highly responsive, mobile friendly, easy-to-use, section 508 compliant website using modern technologies that bridge the gap in access to SUD treatment in Kentucky.

The developed website is hosted via Amazon Web Services to allow for easy sharing of website code and configuration, automatic scalability based on demand, and 99.9% server uptime. During the design and planning phases, the developer provided mockups and outlines that were shared with stakeholders and revised until an effective user interface was developed for both treatment facilities and the public. The website contains two key sections: Administrative and Public. Each section was divided into individual subsections in response to continuous evaluation and testing.

The Public section has five subsections: simple search, search results, resource library, glossary, and information subpages. A simple search feature on the landing page asks 1) who needs help (yourself, friend/family, client/patient); 2) gender of individual; 3) geographical location; 4) type of treatment desired; 5) expected method of payment; 6) whether pregnant, a minor, or LGBTQ. Over 25 additional filters and detailed facility information are displayed after running a search, including but not limited to additional services offered such as mental health, peer support/peer recovery programs, case management services, and information about facility policies pertaining to smoking, prescribed medications, and admissions criteria. Search results are organized by distance from the location entered and include a summary of the SUD treatment facility description, contact information, and an indicator showing availability of treatment with a “last updated” date stamp, along with other attributes viewed by clicking the facility name. Results can be viewed as a summary list or on a map. A searchable resource library that contains documents and other materials related to SUD, treatment, substances, and recovery topics is also available. A glossary of terms was created to assist the public in understanding terminology associated with SUD and treatment; definitions are written at a 6th to 8th grade reading level and linked to terms throughout the website via standard clickable “?” indicators.

The Administrative section for KIPRC and SUD facilities is comprised of four key subsections: Facility Dashboards, Administrative Dashboard, Analytics, and User Management. Facility dashboards allow SUD treatment facilities to manage their profiles including licensure and accreditation, populations served, facility policies, and treatment openings and availability. Managers can add or remove facilities, assign employees to specific facilities to submit regular updates to availability information, and quickly view the status of each facility (if it is published in search results or needs more information, when and by whom it was last updated, and current number of treatment slots available). When a facility has not been updated for a set amount of time (4 days for residential, 30 for outpatient-only), an automatic notification is sent to the manager and any associated employees requesting that they update their availability information as soon as possible. If no updates are posted within 48 h following a second notification, the facility is automatically unlisted from the search results pending new updates. This ensures that the information presented to the public is accurate and up-to-date.

The administrative dashboard is used by KIPRC staff to monitor all information and accounts related to the website. Administrators are able to quickly identify facilities that have pending notifications, have been unpublished due to lack of updates, and have never been reviewed by the associated treatment provider. This system allows managers to proactively identify and contact facilities or provider networks that may need enhanced training or require assistance with the website. The administrative dashboard also provides a dump of database data, which enables rapid assessment and evaluation of all information captured about each facility.

Analytics are available to the public via a separate subsection. The data presented are captured from the provider dashboards and organized to display information about SUD treatment such as treatment availability by location, total numbers of facilities by treatment type and location, treatment availability trends over time, and other information. Additional website utilization information is available through the Google Analytics platform. User management allows administrators and facilities to send invitations to other individuals in their organizations automatically. The system can create an account based on an individual email address and assign the appropriate role and access rights based on the type of access requested.

Quality assurance measures were developed that included weekly communication with the website developers, regular testing of website functionality by KIPRC staff and the development team, continuous database and server performance monitoring, a bug-reporting form for facilities, frequent solicitation of feedback from treatment facilities, and task management software for tracking the status of tasks and bug reports sent to the development team.

### Website usability and focus group testing

Pre- and post-launch qualitative evaluation of the FindHelpNowKY.org website was conducted to assess the flow, content, and ease of use. Through KIPRC’s Cabinet for Health and Family Services (CHFS) bona fide agent status, a CHFS internal email invitation was disseminated to all employees to volunteer for pre-launch FindHelpNowKY.org website usability testing, and focus groups. Approximately 50 employees reviewed and commented on the website, and approximately 25 participated in two simultaneously held focus groups. Each participant was asked to go through the search functionality of FindHelpNowKY.org at least twice, directed and non-directed. To broadly test the capability of FindHelpNowKY.org, each participant was assigned one of six scenarios (developed in collaboration with DBHDID) that represented common overarching themes among individuals seeking SUD treatment. As participants used the site, they were asked to complete a usability testing evaluation form, which included questions about various website features such as search filters, results list of treatment facilities, map function, and profile pages of selected facilities. In the focus groups, some guiding questions included: What did you like best (least) about the website? If you could change one thing about this website, what would it be and why?; What were some of your impressions when you landed on the homepage?; and What stood out to you about the search results?

Eight additional external focus groups were facilitated with individuals who had family members with SUD or were at risk for substance misuse, in partnership with the Kentucky Partnership for Families and Children, Inc. The goal of these focus groups was to listen to their thoughts, processes, and experiences in finding SUD help and treatment for themselves or loved ones. Each focus group session was divided into three parts: treatment-seeking behaviors, usability of the website, and website expectations based on use.

Each focus group participant was asked to discuss their treatment seeking behaviors. They were asked questions such as “What kind of service, treatment, or information resources have you needed in the past? Were you able to find them?” and “What kind of terms or phrases have you used in Internet searches?” On the practical usability of FindHelpNowKY.org, we asked participants to take a moment and think about the experiences they discussed in part 1, then to think of a specific instance for himself or herself or a loved one related to SUD treatment seeking. They were then asked to use the website to find the help they would need in that instance. After participants completed usability testing, we asked them to complete a short online survey to provide feedback on utilization of FindHelpNowKY.org. In the last part of the focus group session, participants were asked, “How does your experience using FindHelpNowKY.org compare to what you hoped to find?” If they did not find what they needed, a follow-up question was asked, “What kind of information or resources would you hope to see on FindHelpNowKY.org?” The goal of this question was to gain insight into expectations and lived experience in searching for SUD treatment and related resources.

Consistent themes emerged from focus groups. Many focus group participants felt that the facility profile descriptive text was lacking, along with use of abbreviations not spelled out. Based on feedback, facility summaries are now mandatory on the profile pages, and abbreviations were removed. In addition, focus groups noticed the lack of accreditation status included in facility profiles; accreditation status is now prominently featured in the “about” profile section. Other changes were made to facility layout pages based on feedback: 1) the position of facility information was modified with the facility’s mapped location, contact information, and hours of operation now at the top of the page; 2) policies are now located in its own section, that was formerly under the information section; 3) facility phone numbers and addresses are now active and clickable across platforms (e.g., computer, smart phone, tablet) to increase accessibility to those utilizing the website; and 4) facility list/map buttons are more prominently featured as an actual button rather than a standard clickable hyperlink. Focus group and usability testing participants felt that the home page was too text heavy, and the background image was not credible, desiring a neutral calming image of “Kentucky”. Based on the detailed feedback, the landing page was reskinned, including the search box and background image. All text was modified using a plain language approach with limited text. The choice of “not sure” was added to the optional search filters; explanatory text was added for some payment options; and a “Pregnant” option was added.

### Compilation of information repository resource documents

Over 400 SUD information repository resources were obtained online in English and Spanish from multiple federal agencies including SAMHSA, National Institute on Drug Abuse (NIDA), Centers for Disease Control and Prevention (CDC), and National Institute for Occupational Safety and Health (NIOSH), among others. SUD document collection focused on 1) SUD conversations related to youths, providers, family, and agencies’ roles; 2) SUD treatment therapy types (including MAT); 3) neonatal abstinence syndrome; 4) SUD related laws and policies (e.g. Casey’s Law and The Good Samaritan Law); 5) SUD recovery support; 6) stigma; and 7) comorbid infectious diseases and medical conditions. Relevant comprehensive documents were incorporated “as is” into the information repository, and approximately 30 additional topics for concise one-page documents targeted to the average layperson at the 4th to 6th grade reading level were identified and developed.

### Evaluation of FindHelpNowKY.org

An evaluation plan was developed for the formative and process evaluation of FindHelpNowKY.org that utilized both qualitative and quantitative evaluation approaches (mixed methods) (Table 2). Website performance measures were developed to measure formative, process, and impact outcomes. Qualitative measures in the formative phase focused on determining the need for the FindHelpNowKY.org website; informal stakeholder interviews were held with two primary healthcare physicians, individuals with SUD and family members, the single source state agency authority for SUD treatment, and the Kentucky ODCP. Quantitative formative measures focused on describing SUD treatment need, SUD treatment facility capacity, and website use capacity.

**Table 2:** Evaluation of FindHelpNowKY.org Intervention

	Intervention Formative Evaluation	Intervention Process Evaluation	Intervention Impact/Outcome Evaluation
Qualitative	- Primary care physician stakeholder interviews to determine website element needs	- SUD treatment facility survey on website function - Primary care provider survey on website usability - Focus group interviews with those in recovery on website usability - Website usability and focus group testing	- SUD treatment facility interviews on client intake questions regarding website use - Completed online website use evaluations - Primary care provider survey of number of referrals based on website use
Quantitative	- Number of SUD treatment facilities in state - Number of individuals with SUD - Number of individuals in SUD treatment - Number of individuals with mobile phones - Number of individuals with computers and internet access	- Monthly tracking of website activity by total number of page views, unique users, search duration (in minutes), user type, SUD treatment facility type, geographic location of user and facilities, gender, insurance status, smoking policy, MAT policy, etc. - Website traffic before and after website promotional blitz (by geographic region of promotional focus)	- Proportion of SUD treatment website searches relative to facility type - Overlay of website user locations by treatment facility type coverage area (30 mile radius) to determine user need vs. SUD treatment facility type capacity - Overlay of website SUD treatment facility location searches by treatment facility type coverage area to determine SUD treatment facility need vs. SUD treatment facility type capacity - Number of SUD treatment facilities with openings tracked over time - Rate of SUD treatment facilities with openings per 100,000 individuals with SUD

Qualitative process evaluation involved website usability testing and focus group results. Quantitative outcome evaluation measures track website usage activity, and compare website traffic before website promotion and after website promotion (by geographic region of website promotion).

Mixed methods outcome evaluation is ongoing, examining both individual level outcomes and treatment capacity outcomes. To access individual outcomes, we are examining quantitative data on changes in number of individuals entering treatment, reduction of waiting times for treatment, and increases in primary care referrals and self-referrals to treatment. Outcome qualitative measures will be based on interviews of SUD treatment facilities on their clients’ referral source for contact with the SUD treatment facility.

In addition, primary care providers will be surveyed on their use of the website in contacting SUD treatment facilities with available openings and securing client entry. Treatment capacity outcome measures will examine quantitative data on SUD treatment facilities searches to identify SUD treatment facility preference, and number and rate of SUD treatment facilities with openings in multiple years to assess SUD treatment capacity over time. GIS maps will be generated to compare SUD treatment availability with SUD treatment needs.

## Results

### FindHelpNowKY.org development and implementation barriers and facilitators

Barriers and facilitators in the development and implementation of the FindHelpNowKY.org website were identified in Table 3. Regarding participation in the website by SUD treatment facilities, staff members first needed to identify the entity(ies) with contact information for the SUD treatment facilities in the state, in addition to the SUD treatment facility list available on the SAMHSA Behavioral Health Treatment Services Locator website. Program staff members were also uncertain of the appropriate persuasive technique that would resonate well with SUD treatment facilities to obtain consent for participation in the website and at times had difficulty identifying the appropriate individual within the provider network with the authority to agree to participate in FindHelpNowKY.org . Last, there was a lack of an onboarding strategy for SUD treatment facility information to be published on the FindHelpNowKY.org website.

**Table 3:** Barriers and Facilitators for FindHelpNowKY.org Development and Implementation

Theme	Barriers/challenges	Facilitators
SUD Treatment Facility FindHelpNowKY.org Participation	- lack of up-to-date contact information on SUD treatment facilities - Uncertain persuasive technique(s) to recruit SUD treatment facility participation in FindHelpNowKY.org updating of available openings on a daily basis - Developing website onboarding strategy for SUD treatment facilities	- ODCP letter to SUD treatment facilities and MAT providers encouraging participation - SUD treatment facility website participation a mandatory condition of ODCP community mental health center funding - Persuasive argument for website participation that rapidly filled SUD openings result in a financial benefit - Introduction of website to SUD treatment facilities by ODCP at Kentucky School of Alcohol and Other Drug Studies - Use of REDCap and adapted SAMHSA SUD treatment facility application to onboard SUD treatment facilities - Strategic recruitment of SUD treatment facilities in conferences
FindHelpNowKY.org Website Development and Implementation Partners	- Partner lack of understanding of FindHelpNowKY.org and their role - Partner processes and priorities did not always align with FindHelpNowKY.org - Varying levels of partner website support - Lack of agreement on naming of website	- Having a partner(s) as a champion to explain website benefit to other partners - Repetitive justification of website benefit to partners
FindHelpNowKY.org Information Repository	- Plethora of information on SUD prevention and treatment - Lack of clear guidance on development of repository information topics and content - Availability of accessible, current, and common reading level appropriate SUD resources	- Regular scheduled meetings to review content and topics - Participation in statewide promotion of website - Distillation of SUD prevention and treatment information using consistent criteria - Regular input from SUD treatment facilities on needed SUD educational resources for clients and general public
FindHelpNowKY.org Website Development	- Inability to hire qualified internal website developer - Lack of website prototype to build upon - Identification of website analytic elements - Incorporation of GIS into website - Development of SUD facility automated notification system	- Hiring of outside website developer based on an issued Request for Proposals (RFP) - Regular scheduled meetings from concept to launched website - Meetings with SUD treatment facilities on desired website analytics that facilitate regulatory reporting to SAMHSA - Feedback from SUD facilities to improve opening availability entry input process and user experience of website
FindHelpNowKY.org Implementation	- Website readiness - Lack of knowledge of website - Lack of statewide promotion of website	- Hiring of advertising agency for statewide promotion of website - Rapid stakeholder feedback on website bugs - Press conference with Governor Bevin to announce website

To overcome these barriers, the Ky ODCP wrote a letter to Alcohol and Other Drug Entity (AODE)/ Behavioral Health Service Organization (BSHO) licensed treatment facilities throughout the state, including all 14 region community mental health centers and to all MAT data waivered physicians (8 h of MAT training is required to apply for a Drug Enforcement Agency waiver in order for physicians to prescribe buprenorphine). For regional mental health center related SUD treatment facilities, the Ky ODCP made FindHelpNowKY.org participation a mandatory condition for community mental health center funding. For non-community mental health center SUD treatment facilities, the potential financial and marketing benefits were discussed emphasizing rapid filling of SUD treatment spots when website users know that there is a SUD treatment opening available.

To onboard SUD treatment facilities, a SUD treatment facility website application was developed in a REDCap database that requested facility information on accreditation; hours of operation; number of hotline numbers; facility type (for profit, non-profit, private, faith-based, community, and block grants received); urban or rural geographical location; forms of accepted payment; referral requirements; admission criteria; available language services; human immunodeficiency virus (HIV)/hepatitis C virus (HCV) screening requirements for treatment; prescribed medication policy; tobacco use policy; gender identity bed assignment policy; population groups served; SUD treatment services offered; outpatient, intensive outpatient, MAT treatment client number maximums; MAT medication availability; additional services (e.g., mental health services, parenting classes, housing assistance, and employment assistance; and others. To raise awareness of the website launch date and to further identify SUD treatment facilities for participation in the website, program staff members attended and delivered presentations at relevant conferences and manned exhibit booths.

The barriers identified in obtaining stakeholder buy-in for the development and implementation of the website included 1) a lack of understanding of the goal of the website and their own role in the development and promotion of the website; 2) varying levels of stakeholder support; and 3) lack of agreement on the naming of the website. Since the website was developed at the university as the bona fide agent of the Department for Public Health, stakeholder processes and priorities did not always align with the goal of the website. To overcome these barriers, a Ky ODCP website champion was identified who could well navigate both academic and governmental processes and priorities, and gain full support for the website by both academia and governmental entities. Repeated discussion of website benefits also helped to gain sustained awareness of the value of the website.

For the FindHelpNowKY.org information repository, barriers identified were that there were hundreds of SUD treatment and prevention documents produced by SAMHSA, NIOSH, NIDA, Partnership for Drug Free Kids, CDC, National Alliance of Advocates For Buprenorphine Treatment, and National Center on Substance Abuse and Child Welfare (NCSACW) that needed to be reviewed and categorized. In addition, the majority of the available documents were at a 12th grade reading level or higher and were not downloadable. Last, staff members lacked clear guidance on the identification of website repository topics and content, and purpose of the information repository. To overcome the barriers, the documents were tagged with keywords and categorized by drug type (fentanyl, benzodiazepines, heroin, hallucinogens, etc.), audience (family and friends, professionals, and people with a substance use disorder), and information type (SUD, general addiction, syringe and needle, and treatment information). Program staff meetings were scheduled on a regular basis, and feedback was solicited from stakeholders.

Barriers to the development of the website commenced with the inability to hire a qualified on-site staff member with website development experience. Another major barrier was the lack of a website prototype to build the FindHelpNowKY.org website with the desired analytic elements, GIS components, and SUD treatment facility automated notification system. These barriers were addressed through the establishment of an external contract with an established local website developer who had experience in building public health related websites with the elements that we required. In addition, to help inform the website analytic capacity and to further gain SUD treatment facility website participation, KIPRC held meetings with individual SUD treatment facilities to identify SAMHSA reporting elements that could be automatically generated from the website for their SAMHSA regulatory reporting purposes. Also, regular feedback was requested and received from SUD treatment facilities on the website availability input process to facilitate quick daily entry of opening availability.

Regarding FindHelpNowKY.org website implementation, a few barriers were identified. First, the website development process began in spring 2017, and many stakeholders were anxious to begin using it as soon as possible. Second, although many stakeholders were aware of the website once it was launched, it was not widely known by the public or by primary care physicians. There was a lack of statewide promotion of the FindHelpNowKY.org website once it went live on February 2, 2018. To overcome these barriers, a press conference was held with KIPRC, Governor Matt Bevin, Public Health Commissioner Jeffrey Howard, and Justice Cabinet Secretary John Tilley to announce the launch of FindHelpNowKY.org. In addition, a public relations advertising agency was hired to promote the website with additional CDC National Center for Injury Prevention and Control (NCIPC) funding. The website was promoted statewide from June 2018–August 2018 in TV spots, radio ads, digital spots, newspapers, and billboards. Program staff also exhibited the website at conferences attended by healthcare and public health professionals. Last, regular feedback is received from website users on website usability and potential bugs that informs the quality assurance process and facilitates continual improvement of the website.

### FindHelpNowKY.org development and implementation processes

The first step of the planning phase for FindHelpNowKY.org was to meet with primary governmental and organizational stakeholders to describe and discuss the original concept for the website, and solicit input into possible SUD treatment facility recruitment strategies, preferred website design and function, potential website and information repository target audiences, and manning of the FindHelpNowKY.org help line that would be embedded within the website (Table 4). The number of website- related staff members and basic employment qualifications of required staff members was also discussed. The state agencies and organizations present in the planning meeting were KIPRC, Kentucky ODCP, Kentucky Department for Public Health, Operation UNITE, and Department for Behavioral Health, Developmental and Intellectual Disabilities (DBHDID).

**Table 4:** Summary of FindHelpNowKY.org Development and Implementation Processes

	Planning phase	Preparation phase	Delivery phase
Recruit FindHelpNowKY.org Concept Development and Implementation Partners	- Identify state agency and organization partners - Meet with senior agency and organization officials to obtain website development buy-in - Obtain CDC NCIPC funding - Develop website formative, process, and outcome evaluation plan	- Provide regular website development updates to partners and request feedback - Partner approval of website design - Pilot test website with partners before website release - Obtain partner agreement on final website layout - Develop website performance metrics	- Promote and disseminate website to development and implementation partners - Obtain website feedback from partners - Collect and analyze website evaluation data
Recruit SUD Treatment Facilities	- Develop criteria for SUD treatment facility listing on website - Develop persuasive argument to obtain SUD treatment facility buy-in - Hire SUD treatment facility recruitment staff	- Meet with DBHDID to obtain SUD treatment facility listings and DBHDID support for website - Develop talking points for SUD treatment facility meetings - Develop SUD treatment facility website application - Identify key contacts for each SUD treatment facility	- Personally meet with SUD treatment facilities - Collect and input SUD treatment facility applications into website
Develop FindHelpNowKY.org Website	- Identify website stakeholders - Hire website developer - Develop concept vision for website layout - Develop requirements for core functionality, including subpages and analytics	- Design website prototype with adequate landing page appearance, GPS location of listed SUD treatment facilities, appropriate jargon, real-time availability measures, website use tracking, and analytics - Hardware testing of website prototype - Develop and pilot test website usability and focus group test questions - Develop glossary of terms	- Conduct website usability and focus group testing with 10 groups - Review usability and focus group testing results and revise website - Develop final website ready for implementation - Continually enhance user experience and functionality based on website use tracking and user feedback - Solicit feedback from SUD treatment facilities on treatment availability functionality
Develop FindHelpNowKY.org Website Information Repository	- Identify website information repository audiences - Familiarize staff with SAMHSA, NIDA, CDC, Justice and Public Safety, and NIOSH SUD treatment and prevention information - Identify and meet with communications partner to develop SUD condensed target audience on-pagers	- Collate SUD information resources from SAMHSA, NIDA CDC, Justice and Public Safety, and NIOSH - Decide on SUD prevention and treatment information topics - Decide SUD prevention and treatment topics for comprehensive documents per audience type - Decide on topics for condensed SUD prevention treatment, and recovery one-pagers and develop one-pagers	- Populate information repository with documents targeted to audience type - Develop and populate information repository with 30 customized one-pagers at a 6th grade reading level
Implement FindHelpNowKY.org	- Identify state recognized champion to promote website - Identification of website launch date based on website development, SUD treatment facility participation status, and partner availability - Identify conferences and meetings to promote website to health and safety professionals	- Coordinate press conference with Governor’s office - Develop press conference agenda with speakers - Register for in-state health and safety professional conferences to promote the website	- Ongoing monitoring, quality assurance, and evaluation of website functionality - Conduct press conference in collaboration with Governor Bevin, Commissioner for Public Health, Justice and Public Safety Cabinet secretary, and director of KIPRC - Promote website at 20 conferences in year of website release - statewide website promotional campaign

Partner participation in FindHelpNowKY.org concept development preparation included regular in-person meetings and teleconferences with partners to solicit feedback on the website during the development stage including the landing page, search results page, information repository page, and facility analytics pages. The website was usability-tested in 10 focus groups total before and during the official launch, and feedback was used to improve all pages within the website in the 2-month timeframe. During the delivery phase, the website is actively promoted through implementation partners, and is evaluated using 1) an available online website evaluation form embedded within the website; 2) website user data; and 3) website advertising impressions.

The planning meeting to recruit SUD treatment facilities resulted in the Kentucky ODCP office including registration and use of FindHelpNowKY.org as a condition of funding awarded to regional community mental health center SUD treatment facilities. Community mental health center- affiliated SUD treatment facilities account for approximately 24% of facility enrollment in the FindHelpNowKY.org website. In addition, the financial benefits of rapid filling of SUD treatment slots by individuals with SUD by expected insurance coverage was chosen as the preferred strategy to recruit private and other public SUD treatment facility participation in the website.

During the preparation phase, staff members met with DBHDID to obtain SUD facility contact information and their support for the website. Talking points were developed for the SUD treatment facility meetings that included how to participate, expectations for updating information, role of a facility director and facility update manager, benefits to the facility such as usefulness to staff in referral process, increased visibility to community through marketing of the website, etc. The REDCap SUD facility application was developed based on input from DBHDID personnel and SUD treatment facilities (mainly community mental health center facilities), and the AODE/BHSO licensure list was obtained from DBHDID.

For the delivery phase, staff members met in-person with 105 SUD treatment providers that represented 257 facilities from January 2017–February 2018. During the in-person meetings, facility applications were distributed and completed applications were entered manually into the FindHelpNowKY.org website. To maintain an up-to-date inventory of SUD treatment facilities, SUD treatment facilities update their availability on a daily basis. The updating process is facilitated through a login and password, followed by easy toggling of the number of treatment slots. A process was established for notification of SUD treatment facilities that have not updated their availability within a specified amount of time depending on the type of treatment offered. For residential facilities, the first notification is sent after 4 days without an update. For outpatient-only facilities (including MAT physicians, detox, and intensive outpatient) the first notification is sent after 30 days with no updates. First, the facility will receive an automated email stating they need to update their availability as soon as possible, or risk being unpublished after 4 days. Another notice is sent 48 h later indicating that they will be removed from the search results in 2 days; 48 h after the second notification, the facility is unpublished from the website. If the facility wishes to be republished on the website, the facility needs to submit a new update or indicate “no changes”, then click the button to request publication. FindHelpNowKY administrators are automatically notified, review, and approve or deny the publication request barring any problems with the information submitted. Staff members follow up with unpublished facility listings via emails and phone calls weekly.

Planning for the development of the website first involved identifying relevant website development stakeholders such as the Department for Public Health, primary healthcare providers, SUD treatment facilities, and DBHDID, then developing a shared vision for the FindHelpNowKY.org concept. The website developer was hired through an official Request for Applications (RFA) competitive process. Through informal interviews with SUD treatment facilities, and families and friends of those with SUD, requirements for website core functionality were developed. During the preparation phase, a website prototype was developed that contained landing page elements, GIS location of facilities, and website analytics basics. There was hardware testing of the website in the CHFS focus groups and internal testing at KIPRC instructing participants to visit specific pages on work computers and mobile phones, to test browser and device software code in different environments. Project staff members developed a glossary of terms for the website with definitions written at a 6th to 8th grade reading level. During the delivery phase, 10 focus group total tested the website for usability, functionality, and appearance; results were reviewed and the launched website incorporated website usability testing results. Website use is tracked, and feedback is regularly solicited from SUD treatment facilities.

During the planning phase for the information repository, target audiences were identified, and project staff members familiarized themselves with available SUD treatment and prevention information. A communications partner was identified to develop condensed one-pagers for the repository. All available SUD treatment and prevention information source documents were compiled and categorized, and topics for one-pagers were identified during the preparation phase. For the implementation phase, the website was populated with the categorized comprehensive informational documents and with the developed condensed one-page SUD prevention and treatment sheets at a 6th to 8th grade reading level.

Ky ODCP was identified as the state champion for the FindHelpNowKY.org website implementation-planning phase, and a viable launch date was set based on website development progress, SUD treatment facility participation numbers, and partner availability for the press conference. During the preparation phase, the launch date press conference was coordinated with the Governor’s office, Department for Public Health, and ODCP in the Justice Cabinet, and a press conference agenda was developed. In addition, program staff members registered for multiple health and safety professional conferences for oral presentations, and for exhibitions of the website. The delivery phase of FindHelpnowKY.org involves ongoing project staff monitoring of website functionality, regular submission of bug reports and feature enhancements to website developers, and SUD treatment facility participation. To promote the website, the press conference was conducted, 20 conference presentations of the website will be held in 2018, and a statewide promotional campaign is ongoing over the summer of 2018.

### FindHelpNowKY.org evaluation results

Related to the first short-term desired outcome of the website implementation, as of October 15, 2018, the website is indexing 503 treatment facilities and MAT DATA-waivered physicians. More specifically, approximately 83% (463/558) of SUD treatment facilities are indexed on the website. In addition, 8% (40/485) of MAT data waivered prescribing physicians are indexed on the website. There have been over 29,000 visitors (68% unique visitors, and 32% repeat visitors), 160,000 page views, and over 30,000 unique searches. Since the statewide website promotional campaign began June 1, 2018, website use has increased by approximately 1500 new visitors per week. The most common website query is from a friend or family member seeking long-term residential or outpatient treatment facilities that accept Medicaid or Medicare. The average website user spends approximately 7 min on the site and views 3.8 pages. Approximately 38% of website users accessed the website through a mobile phone, 55% accessed through a desktop, and 7% accessed through a tablet. There were 1191 visitors to the resource library.

In addressing our second short-term outcome to expand coverage area of the KY HELP line, Operation UNITE’s statewide KY HELP line has connected individuals with SUD to additional support services that the website cannot offer. The KY HELP line is averaging approximately 280 statewide calls monthly; a treatment and referral specialist provides immediate help with SUD treatment screening and referral, and answers additional personal questions related to SUD treatment and recovery [23]. To assess our third short-term outcome to increase healthcare professional use of FindHelpNowKY.org, 1934 healthcare professionals searched the website from January 15, 2018 to October 15, 2018; continued promotion of the website to healthcare professionals is needed. Related to our last desired short-term outcomes to increase collaboration among partners and to increase alignment among state SUD stakeholders, the website has leveraged new partnerships; for example, the team is currently collaborating with DBHDID on the inclusion of SUD recovery centers in FindHelpNowKY.org.

Analysis of treatment facility and website user data provides insight into SUD treatment availability vs. SUD treatment need in the FindHelpNowKY.org website. As of October 15, 2018, 248 facilities reported being in urban locations and 163 reported their location type as rural (about 1.5/1 ratio), and 92 facilities did not select an option; approximately 70% of the 29,000 website visitors were from urban locations. Of the 503 total SUD treatment facilities, 289 offer treatment for comorbid mental health disorders; there were 298 website searches for comorbid mental health treatment. There are 192 (38.2%) facilities that provide SUD treatment to adolescents and 311 (61.8%) that treat adults; approximately 740 searches were conducted for adolescent treatment. Facilities that treat pregnant females comprise 488 of facilities listed, with 177 offering treatment to pregnant adolescents and 309 treating adults. Of these facilities, 142 have indicated that they only treat pregnant adults, and only 10 treat pregnant adolescents exclusively. Approximately 720 searches were conducted for pregnant females, but limitations with the current Google Analytics configuration prevent accurate counting of searches for pregnant adolescents.

In August, 2018, intake specialists and managers from six SUD treatment facilities listed on the website were contacted to informally assess if and how the FindHelpNowKy.org was being utilized. Specifically, facilities were asked if: 1) clients cited the website as a means of locating their facility; 2) facility staff used the website as a resource to assist or refer clients elsewhere; and 3) general feedback about the website. At that time, 4/6 (66.6%) facilities indicated that they used the website as a resource for referrals or shared the website with a caller when they could not accept a client for treatment for various reasons (e.g. no openings, client’s insurance, client’s location). None of the facilities indicated clients citing the website as a source for locating their facility during the intake process or a later time. In October, 2018, follow-up calls were made to all six facilities, and 4/6 (66.6%) shared that staff utilized the website as a resource for referrals, shared the website with a caller, or transferred a caller to the KY Help Line and 1/6 (16.6%) (a larger community mental health center) indicated that callers specifically mentioned www.FindHelpNowKy.org during the intake process or a later time.

## Discussion

The establishment of the FindHelpNowKy.org website is an additional resource for rapidly matching individuals with SUDs to SUD treatment facilities with current openings that are based on the individual’s needs and preferences. SUD treatment facilities are now accustomed to daily updating of their opening availability, and new SUD treatment facilities are requesting publishing on the website, including facilities from neighboring states. From February 2018 to August 2018, SUD treatment facility website listings of available openings increased from 5% of SUD facilities with updated availability in February 2018 to 95% of listed SUD treatment facilities with updated availability in August 2018.

Multidisciplinary partnerships among numerous governmental agencies, academia, and organizations, and a website champion were integral to the establishment of the website because of their relevant knowledge, experience, and diplomacy skills. Without the buy-in and support of DBHDID, ODCP, and Operation UNITE, the website could not be developed. DBHDID was integral in identifying SUD treatment facilities and establishing SUD treatment facility website inclusion criteria. ODCP was indispensable in gaining SUD treatment facility and MAT provider website participation through the website participation contingency included in regional mental health center funding awards, and through ODCP letters to MAT providers endorsing participation in the FindHelpNowKY.org website. Operation UNITE included their statewide help call center line within the FindHelpNowKY.org website where treatment and referral specialists can provide immediate help with SUD treatment resources and questions.

Evaluation of the FindHelpNowKY.org website is ongoing, guided by both quantitative and qualitative data collection and analyses, and use of evaluation results, to improve website functionality and use. The development process evaluation has yielded actionable information on SUD treatment capacity in Kentucky. After the website promotional campaign has been implemented for 6 months in December 2018, SUD treatment facilities will be interviewed on client referral source to their facility (to assess whether FindHelpNowKY.org was the referral source), and primary healthcare providers will be surveyed on SUD treatment facility referrals based on FindHelpNowKY.org website use.

Online website user evaluations, as well as website user and SUD treatment facility statistics help identify 1) state SUD treatment facility capacity (by SUD treatment facility type) vs. SUD treatment need in the state; 2) SUD treatment providers not licensed by the state; and 3) engagement of DATA-waivered providers. In addition, since the website displays SUD treatment facility services such as housing and employment assistance, provision of MAT, and availability of co-treatment for health conditions such as mental health, eating disorder, and diabetes treatment etc., the website is a useful tool to assess the need and capacity for concurrent treatment of specific health conditions.

FindHelpNowKY.org is a model website that be used by other states who want to visually display all of their respective SUD treatment facilities that have available openings, and other states can use FindHelpNowKY.org when their residents are seeking SUD treatment in Kentucky. Interest has been expressed by other states for use of the FindHelpNowKY.org website hub for their residents’ timely access to SUD treatment. Program staff are currently developing technical assistance guidance for states on how to establish a FindHelpNow website and are providing in-person technical assistance. KIPRC’s Kentucky Drug Overdose Prevention program is obtaining the FindHelpNow.org domain for states to use as the common website platform, and the SUD treatment facility application will be made available for all interested states to utilize.

## Conclusions

FindHelpNowKY.org has filled a need for timely access to facilities with available SUD treatment openings in Kentucky. The website may be a valuable resource for health professionals, including primary healthcare providers and social workers, which can enhance clinical workflow by reducing necessary staff time in conducting phone searches and website searches for SUD treatment facilities that are accepting patients and clients at that moment. FindHelpNowKY.org is a promising tool for assessing current statewide SUD treatment capacity vs. SUD treatment need, and for determining the statewide need for attendant health services. Technical assistance and the website platform will be made available to other states to establish their respective FindHelpNow websites.

## Abbreviations

AODE: Alcohol and other drug entity
BSHO: Behavioral Health Service Organization
CDC: Centers for Disease Control and Prevention
CHFS: Cabinet for Health and Family Services
DBHDID: Department for Behavioral Health, Developmental and Intellectual Disabilities
ED: Emergency department
GIS: Geographic information system
HCV: Hepatitis C virus
HIV: Human immunodeficiency virus
KIPRC: Kentucky Injury Prevention and Research Center
MAT: Medication-assisted treatment
NCIPC: National Center for Injury Prevention and Control
NCSACW: National Center on Substance Abuse and Child Welfare
NIDA: National Institute on Drug Abuse
NIOSH: National Institute for Occupational Safety and Health
ODCP: Office of Drug Control Policy
Operation UNITE: Operation Unlawful Narcotics Investigations, Treatment and Education
RFA: Request for Applications
SAMHSA: Substance Abuse and Mental Health Services Administration
SUD: Substance use disorder
TEDS-D: Treatment episode datasets-discharge
